# Analysis of sleep disorders and their influencing factors in patients with ankylosing spondylitis

**DOI:** 10.1371/journal.pone.0323324

**Published:** 2025-05-13

**Authors:** Xihong Ying, Qiuyan Zhao, Yi Wu, Shasha Deng, Qing Ma, Ronghua Fang

**Affiliations:** 1 General Practice Ward/International Medical Center Ward, General Practice Medical Center, West China Hospital, Sichuan University/West China School of Nursing, Sichuan University, Chengdu, China; 2 General Practice Ward/International Medical Center Ward, General Practice Medical Center, West China Hospital, Sichuan University, Chengdu, China; Prime Hospital LLC, UNITED ARAB EMIRATES

## Abstract

**Objectives:**

Sleep disorders are a common symptom in Ankylosing Spondylitis (AS) patients. In this cross-sectional study, we aimed to understand the current status of sleep disorders in AS patients and to analyze potential factors influencing sleep disorders.

**Methods:**

A total of 205 AS patients were recruited in the survey. The content included the self-designed demographic data questionnaire, The MOS 36-Item Short Form Health Survey (SF-36), Visual Analogue Scale (VAS), Multidimensional Fatigue Inventory (MF-20), Self-Rating Anxiety and Depression Scale, Pittsburgh Sleep Quality Index questionnaire (PSQI), Bath Ankylosing Spondylitis Disease Activity Index (BASDAI), Bath Ankylosing Spondylitis Functional Index (BASFI) and Bath Ankylosing Spondylitis Metrology Index (BASMI). These data were analyzed using chi-square test, independent sample t-test, Mann-Whitney U test, Pearson correlation analysis, single-factor linear regression analysis, and multiple linear stepwise regression analysis.

**Results:**

The results showed that the average sleep total score was 8.82 ± 4.146, and the prevalence of sleep disorders was approximately 66.8% in AS patients. Age (F = 29.710, P < 0.001), disease duration (F = 13.025, P < 0.001), anxiety (F = 36.060, P < 0.001), depression (F = 11.808, P < 0.001), and quality of life (t = 6.665, P < 0.001) significantly impacted the sleep total score. Pearson correlation analysis revealed a significant negative correlation between SF-36 total score and sleep total score (r = ‒0.449, P < 0.01), while positive correlations were observed for VAS score, fatigue, anxiety, depression, BASDAI, BASFI, BASMI, age, and disease duration (all P < 0.01). Multivariate analysis showed that age, disease duration, nocturnal pain VAS score, total back pain VAS score, peripheral joint pain VAS score, total fatigue score, total anxiety score, and BASMI total score significantly predicted sleep total score (R² = 0.755, F = 45.334, P < 0.001).

**Conclusion:**

These findings suggest that medical professionals should pay increased attention to the observed associations between sleep disorders and clinical factors in AS patients, and consider implementing targeted interventions to address sleep-related issues.

## Introduction

Ankylosing spondylitis (AS) is a complex chronic autoimmune inflammatory disease, its primary characteristics include chronic back pain, stiffness in the back, and progressive impairment of spinal mobility [1,2], which can lead to vertebral fusion in severe cases [3]. AS can occur at any age, with the most common onset starting between the ages of 20 and 30 [2,4]. The epidemiological survey results have shown a global prevalence of approximately 0.2% to 1.2%, with a male-to-female ratio of approximately 3:1 [5,6]. When joint ankylosis occurs, patients may experience issues such as anxiety, depression, and sleep disorders due to restricted mobility and pain, seriously impacting their quality of life.

Sleep disorders refer to manifestations of disturbances in the normal rhythmic alternation between sleep and wakefulness. The research results indicated that sleep disorders were common symptoms of cardiovascular diseases, neurodegenerative diseases, and cancer, which could increase mortality rates and lead to a significant medical burden [7,8]. An increasing number of research clarified that AS patients had various sleep problems, including poor sleep quality, falling asleep insomnia, difficulty awakening, and obstructive sleep apnoea syndrome (OSAS) [9]. Sleep issues are common not only in AS but also in other rheumatic diseases like rheumatoid arthritis (RA) and systemic lupus erythematosus (SLE) [10,11]. For instance, RA patients often experience sleep disorders, including poor sleep quality and insomnia, which are strongly associated with disease activity, pain severity, and psychological distress [11–13]. Given the shared mechanisms, such as chronic inflammation, pain, and psychological burden), further research is essential to better understand and manage sleep disorders across rheumatic diseases. It is noteworthy that the prevalence of sleep disorders in AS patients is twice that of the general population [14]. Walsh et al. reported on comorbidity burden using a large administrative claims database in the United States. The analysis revealed a significantly higher prevalence of OSAS in AS patients compared to the control group [15]. Additionally, another cross-sectional study from Sweden obtained similar results, with a prevalence of approximately 9% for OSAS in AS patients [16]. Similar patterns have been observed in RA, where the prevalence of OSAS is also significantly higher than in the general population [17]. These findings suggest that sleep disorders may be a common comorbidity in rheumatic diseases, driven by shared pathogenic mechanisms such as chronic inflammation and immune dysregulation. Given these findings, gaining a better understanding of the factors influencing sleep disorders in AS is crucial for developing targeted AS interventions and improving quality of life. The research results have indicated that functional ability (BASFI, BASMI, BAS-G, and BAS-G1) serves as a predictive indicator of poor sleep in AS patients, and poor functional status is associated with poor sleep quality, longer sleep latency, shorter sleep duration, and poorer sleep efficiency [18]. Furthermore, Sariyildiz et al. showed a positive correlation between sleep disorders and poor quality of life, with lower sleep quality closely related to quality of life [19]. In addition to functional ability, other factors such as HLA-B27 positivity, BASDAI, depressive symptoms, and disease duration also play significant roles in predicting sleep disturbances in AS patients [20].

These findings have suggested that factors associated with sleep disorders in AS patients are relatively complex. Therefore, this study primarily investigated AS patients, systematically understood the status of sleep disorders in Chinese AS patients using some methods such as questionnaires and internationally recognized scales. Furthermore, we comprehensively identified relevant factors, and aimed to provide reliable clinical basis for further improving the quality of life of AS patients and enhancing the prevention and treatment of psychological health in AS patients.

## Materials and methods

### Study design

The reporting of this study conforms to the STROBE guidelines [21]. This was a cross-sectional study that collected data from AS patients who received treatment at the outpatient and special outpatient departments of West China Hospital, Sichuan University, between June 2023 and June 2024. A self-designed general questionnaire was used to conduct the survey. Paper-based questionnaires were administered with the assistance of trained investigators. To ensure the smooth completion of the questionnaires, standardized instructions were provided to help elderly participants understand the content. After completion, the questionnaires were collected on-site and checked for completeness. If any missing or incomplete answers are found, the questionnaire will be returned to the participants for supplementation. If any questions were left unanswered or incomplete, the questionnaires were returned to the participants for revision (S1 File).

### Participants

Patients were recruited using convenience sampling. Specifically, all eligible AS patients who visited the outpatient and special outpatient departments during the study period were consecutively invited to participate. A total of 209 AS patients who met the inclusion and exclusion criteria were enrolled. Inclusion criteria include: (1) Meeting the 1984 revised New York classification criteria for AS [22]; (2) Patients voluntarily participating in this study after being informed about the research; (3) Voluntarily signing an informed consent form. Exclusion criteria include: (1) Having other rheumatic immune system diseases, mental illnesses, as well as severe heart, liver, kidney dysfunction, and blood system disorders; (2) Severe cognitive impairment or being unable to cooperate with the study; (3) Received psychological or sleep-related medication treatment in the past 3 months; (4) Inability to participate in the trial due to other health issues; (5) Inability to sign the informed consent form or participate in questionnaire surveys and scale assessments; (6) Having a history of dependence on psychotropic drugs or being diagnosed with a mental disorder by a doctor.

### Consent to participate

All participants in this study were fully informed about the purpose and procedures of the research. Verbal/written consent was obtained from each participant before collecting the data. Participants were assured that their responses would remain confidential and used solely for research purposes.

### Data collection

Basic information from AS patients were collected (including Gender, age, height, occupation, marital status, education level, disease course, smoking history etc.). In addition, the internationally standardized scales were utilized to assess pain, BASDAI, BASFI, BASMI, sleep quality, life quality, fatigue, anxiety, and depression in AS patients. Data collection for this cross-sectional study was finalized on 10/07/2024 after all questionnaire responses were entered into the database for analysis.

The sleep quality assessment of AS patients in the past month was conducted using the Pittsburgh Sleep Quality Index (PSQI) [23–25]. The score for each item ranges from 0 to 3 points, and the total score of the seven components is the PSQI score with a range from 0 to 21. Sleep quality is inversely proportional to the scores, the higher the scores, the worse the sleep quality.

The fatigue assessment of AS patients in the past two weeks was conducted using the Multidimensional Fatigue Inventory (MFI-20) [26,27]. The total score ranges from 20 to 100, with higher scores indicating more severe fatigue symptoms. The MOS 36-Item Short Form Health Survey (SF-36) is used to evaluate the quality of life of AS patients over the past month. The scale comprises 36 questions, and patients respond to them based on their circumstances. The total score ranges from 0 to 100, with higher scores indicating a better quality of life [28,29]. The pain assessment of AS patients over the past week was conducted using the Visual Analog Scale (VAS) [30,31], including three items: nocturnal pain, total back pain, and peripheral joint pain. The recording range scale was from 0 cm (none) to 10 cm (very severe), with a total score range of 1–10. The higher the score, the more severe the pain.

The Bath Ankylosing Spondylitis Disease Activity Index (BASDAI) was used to evaluate the disease activity of AS patients over the past week. The overall score is calculated, and higher scores indicate more active disease. Generally, a total score greater than 4 suggests disease activity. The Bath Ankylosing Spondylitis Functional Index (BASFI) was used to evaluate the functional status of AS patients. The total score ranges from 0 to 10, with higher the score indicating a worse the functional level of AS patients. The Bath Ankylosing Spondylitis Metrology Index (BASMI) is used to evaluate the mobility of the spine and hip joints in AS patients, including tragus to wall distance (cm), cervical rotation angle, lumbar side flexion mobility (cm), lumbar mobility (revised Schober’s test), and intermalleolar distance. The total score ranges from 0 to 10, with higher scores indicating poorer spine mobility.

The evaluation of anxiety status in AS patients was conducted using Zung Self-Rating Anxiety Scale (SAS) [32], A standardized scoring algorithm was employed to define the anxiety status, with the raw score derived from the sum of scores for all items. The total score ranges from 20 to 80, with higher scores indicating greater level of anxiety. The Self-Rating Depression Scale (SDS) is a widely used clinical tool for screening depression [33,34], primarily used to evaluate the depression symptoms and severity of AS patients over the past week. The raw score was the sum of scores for all items, ranging from 20 to 80, with higher scores indicating a more severe level of depression [35–37].

### Ethical principles

The research protocol, amendments, and informed consent form for this experiment have been approved by the Biomedical Ethics Review Committee of West China Hospital, Sichuan University [Ethical approval number: 20 2023 Review (No. 1040)].

### Sample size calculation

The sample size was estimated based on the requirements for multiple regression analysis. Using G*Power [38], with an effect size of f^2^ = 0.1(weak to medium), 16 predictors, a power of 0.8, and a significance level of 0.05, the minimum required sample size was calculated as 183. Accounting for a 20% dropout rate, the target sample size was 220. A total of 209 AS patients were recruited, which was close to the recommended sample size and provided sufficient statistical power for the planned analyses.

### Statistical analysis

All data were analyzed using SPSS 26.0 statistical software. The normal distribution was expressed by mean ± SD, while non-normal distribution was expressed by median (interquartile range), and count data were presented as rates or percentage. The differential tests between the sleep disorder group and the non-sleep disorder group of AS patients were tested using chi-square test, independent sample t-test, and Mann Whitney U test. Pearson correlation analysis was employed for correlation analysis. Disease-related factors (pain, fatigue, quality of life, BASDAI, BASFI, BASMI) that may contribute to sleep disorders in AS patients were treated as multiple independent variables, with sleep disorders as the dependent variable for statistical analysis. Single-factor linear regression analysis was conducted initially, followed by multiple linear stepwise regression analysis. A significance level of P < 0.05 was considered statistically significant.

## Results

This study distributed a total of 209 questionnaires from June 2023 to June 2024, collecting data from AS patients. Among them, 3 patients did not complete the questionnaire survey, and 1 patient discontinued the questionnaire survey. Ultimately, 205 questionnaires were completed, resulting in an effective response rate of 98.1%.

### Patient characteristics

The characteristics of 205 AS patients were summarized in Table 1. The patients were aged between 15 and 66, with an average age of 33.60 ± 11.20 years. Among them, 67.3% of patients were male, 93.7% were of Han nationality. Of the participants, 54.6% were only children, 80.0% were employed, and 12.2% were students. 65.3% had received more than 12 years of education, 71.2% lived in nuclear families, and 25.4% lived in extended families. 93.2% of the patients had a per capita family income monthly of over 2000 yuan, and the majority of patients had a good quality of life (n = 188, 91.7%). The duration of illness among patients was fairly balanced, with 36.6% having an illness duration of 3–5 years and 34.6% having it for more than 5 years. 85.9% of patients showed no signs of anxiety, 42.0% had no depression, and 32.7% experienced mild depression. There were 190 HLA-B27 serum positive patients (92.70%) and 15 HLA-B27 serum negative patients (7.30%), as shown in **Table 1**.

**Table 1:** Demographic and clinical characteristics of AS patients.

Characteristics	AS patients (N = 205)	Percentage (%)
Age, years		100
<18	15	7.3
18-60	179	87.3
>60	11	5.4
Gender		100
Male	138	67.3
Female	67	32.7
Nation		100
The Han nationality	192	93.7
Minority nationality	13	6.3
Family ties		100
One-child family	112	54.6
Not one-child family	93	45.4
Employment		100
Employed	164	80.0
Student	25	12.2
Unemployed	16	7.8
Family structure		100
Nuclear family	146	71.2
Extended family	52	25.4
Reorganization/single parent families	7	3.4
Education level, years		100
<9	28	13.7
9-12	43	21.0
12-16	114	55.6
>16	20	9.7
Per capita family income monthly, yuan		100
≤2000	14	6.8
>2000	191	93.2
Disease duration, years		100
＜1	7	3.4
1-3	52	25.4
3-5	75	36.6
>5	71	34.6
Anxiety rating		100
No anxiety	176	85.9
Mild anxiety	14	6.8
Moderate Anxiety	9	4.4
Severe anxiety	6	2.9
Depression rating		100
No depression	86	42.0
Mild depression	67	32.7
Moderate depression	33	16.1
Severe depression	19	9.2
Life quality rating		100
General	17	8.3
Good	188	91.7
HLA-B27 Gene Positive Negative	190 15	100 92.7 7.3

AS, ankylosing spondylitis; HLA-B27, human leukocyte antigen B27. Data are presented as number (percentage) unless otherwise specified. Percentages may not sum to 100% due to rounding.

### Single-factor analysis of variance for sleep disorders in AS patients

This study used the sleep total score of AS patients as the dependent variable, and general demographic information and clinical parameters of patients as independent variables. SPSS 26.0 software was used to conduct single-factor analysis between the sleep total score and the the patients' general social demographic data and clinical parameters.The Shapiro-Wilk test was used to assess the normal distribution of the sleep total score, and it was found to be in accordance with a normal distribution (P > 0.05). Therefore, t-tests or analysis of variance was used. The statistical results were shown in **Table 2**. In social demographic information, age (F = 29.710, P < 0.001), disease duration (F = 13.025, P < 0.001), and gender (t = -2.251, P = 0.020), as well as clinical variables including anxiety (F = 36.060, P < 0.001), depression (F = 11.808, P < 0.001), quality of life (t = 6.665, P < 0.001) and HLA-B27 gene (t = -8.784, P < 0.001) all showed significant differences in sleep total score. Specifically, age, disease duration, anxiety, and depression were significantly correlated with higher sleep total score, while quality of life was significantly associated with lower sleep total score. However, in social demographic information, nationality (P = 0.551), family ties (P = 0.262), per capita family income monthly (P = 0.443), family structure (P = 0.548), and education level (P = 0.339) showed no significant differences in the sleep total score among AS patients.

**Table 2:** Single-factor analysis of variance for sleep disorders in AS patients (x ± s).

Variable	Total score of sleep (point)	Analysis methods		P
Age, years				
< 18	4.800 ± 1.935	F = 29.710		＜0.001
18-60	8.721 ± 3.754			
>60	15.909 ± 3.859			
Disease duration, years				
< 1	3.714 ± 0.488	F = 13.025		＜0.001
1-3	7.731 ± 3.390			
3-5	8.133 ± 4.111			
> 5	10.845 ± 3.959			
Family structure				
Core family	8.630 ± 4.136	F = 0.603		0.548
Extended family	9.365 ± 3.951			
Reorganization/single parent families	8.714 ± 5.908			
Education level, years				
＜9	7.536 ± 4.141	F = 1.128		0.339
9-12	8.767 ± 4.099			
12-16	9.300 ± 3.701			
>16				
Anxiety rating				
No anxiety	7.881 ± 3.408	F = 36.060		＜0.001
Mild anxiety	12.571 ± 3.736			
Moderate Anxiety	15.222 ± 2.728			
Severe anxiety	18.000 ± 1.095			
Depression rating				
No depression	7.512± 3.395	F = 11.808		＜0.001
Mild depression	8.672 ± 3.686			
Moderate depression	10.182 ± 4.620			
Severe depression	12.895 ± 4.841			
Gender				
Male		8.326 ± 3.880	t = -2.251	0.020
Female		9.836 ± 4.508		
Nation				
The Han nationality		8.865 ± 4.154	t = 0.597	0.551
Others		8.154 ± 4.120		
Family ties				
One-child family		9.116 ± 4.187	t = 1.125	0.262
Not one-child family		8.462 ± 4.090		
Per capita family income monthly, yuan				
≤ 2000		9.643 ± 4.668	t = 0.765	0.443
> 2000		8.759 ± 4.112		
Life quality rating				
General		14.647 ± 4.030	t = 6.665	＜0.001
Good		8.293 ± 3.741		
HLA-B27 Gene Positive Negative		9.140 ± 4.120 4.800 ± 1.500	-8.784	＜0.001

Data are presented as mean ± standard deviation (x̄ ± s). Analysis methods include one-way ANOVA (F) for categorical variables with more than two groups and independent samples t-test (t) for binary variables. *P < 0.05, **P < 0.01, ***P < 0.001.

### The impact of sleep disorder levels on clinical parameters in AS patients

In this study, the sleep total score was divided into four groups based on the level of sleep disorders (no sleep disorders, mild sleep disorders, moderate sleep disorders, severe sleep disorders). The analysis results (Table 3) showed significant differences among different sleep disorders severity groups in the SF-36 scale (Chinese version) total score (F = 28.688, P < 0.001), nocturnal pain VAS score (F = 34.587, P < 0.001), total back pain VAS score (F = 53.894, P < 0.001), peripheral joint pain VAS score (F = 58.364, P < 0.001), total fatigue score (F = 22.922, P < 0.001), total depression score (F = 15.488, P < 0.001), BASDAI score (F = 18.386, P < 0.001), BASFI score (F = 33.820, P < 0.001), and BASMI total score (F = 53.644, P < 0.001) for AS patients. As the severity of sleep disorders increased, the pain intensity in the nocturnal pain, total back pain, and peripheral joint pain became more pronounced, the severity of fatigue and depression also increased, along with higher BASDAI score, BASFI score, and BASMI total score.

**Table 3:** Single-factor analysis of variance between general social demographic data and clinical parameters and the degree of sleep disorders.

	Sleep rating (Mean ± SD)				F	P
	Satisfactory (n = 48)	Moderate (n = 96)	Adequate (n = 43)	Unsatisfactory (n = 18)		
SF-36 total scores	126.39 ± 11.97	124.18 ± 14.49	118.80 ± 16.50^ab^	92.82 ± 8.6^abc^	28.69	<0.001
Nocturnal pain (VAS)	1.50 ± 1.69^bcd^	2.527 ± 1.99 cd	4.22 ± 2.68^d^	6.72 ± 1.64	34.59	<0.001
Total back pain (VAS)	0.95 ± 1.19^bcd^	2.28 ± 1.97 cd	3.96 ± 2.45^d^	6.98 ± 0.82	53.89	<0.001
Peripheral joint pain (VAS)	1.01 ± 1.06 cd	1.40 ± 1.49 cd	3.97 ± 2.77^d^	6.03 ± 1.1	58.36	<0.001
Fatigue total score	49.12 ± 9.19^bcd^	55.66 ± 5.68 cd	59.07 ± 4.57	59.44 ± 3.00	22.92	<0.001
Depression total score	30.21 ± 7.61 cd	32.65 ± 6.74 cd	36.19 ± 8.36^d^	43.11 ± 7.94	15.49	<0.001
BASDAI	2.08 ± 1.32^d^	2.19 ± 1.44^d^	2.46 ± 1.47^d^	4.71 ± 0.88	18.39	<0.001
BASFI	1.43 ± 1.10 cd	1.79 ± 1.36^d^	2.05 ± 1.68^d^	5.06 ± 1.11	33.820	<0.001
BASMI total score	1.48 ± 2.11 cd	1.57 ± 2.06 cd	4.54 ± 1.91^d^	7.17 ± 2.50	53.644	<0.001

SF-36, 36-Item Short Form Survey; VAS, Visual Analog Scale; BASDAI, Bath Ankylosing Spondylitis Disease Activity Index; BASFI, Bath Ankylosing Spondylitis Functional Index; BASMI, Bath Ankylosing Spondylitis Metrology Index. a: Compared with the group with satisfactory sleep quality, P < 0.001; b: compared with the group with moderate sleep quality, P < 0.001; c: compared with the group with adequate sleep quality, P < 0.001; d: compared with the group with unsatisfactory sleep quality, P < 0.001.

### Pearson correlation analysis results

**Fig 1:**
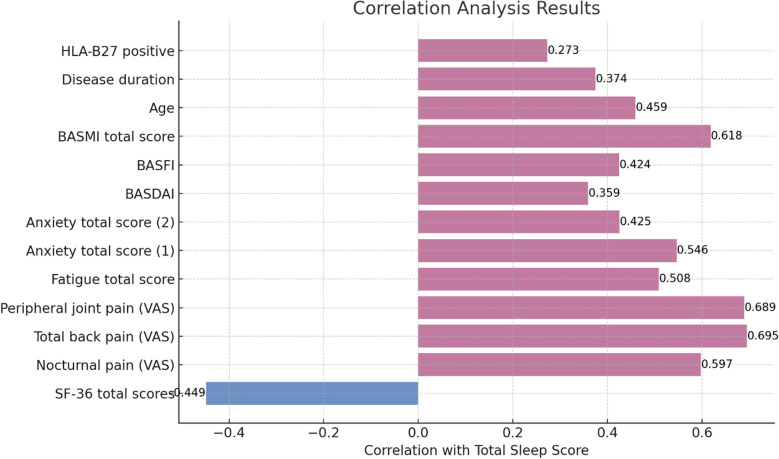
Pearson correlation analysis in AS patients. Significant negative correlation between SF-36 total score and sleep total score (P < 0.01). Positive correlations (P < 0.01) observed for VAS score, fatigue, anxiety, depression, BASDAI, BASFI, BASMI, age, disease duration, HLA-B27 positivity, and sleep total score.

This study conducted Pearson correlation analyses between the SF-36 scale total score, VAS score, total fatigue score, total anxiety score, total depression score, BASDAI score, BASFI score, BASMI total score, age, disease duration, and sleep total score in AS patients. The results showed a significant negative correlation between the SF-36 scale total score and sleep total score (r = -0.449, P < 0.01). While the VAS score, total fatigue score, total anxiety score, total depression score, BASDAI score, BASFI score, BASMI total score, age, disease duration, HLA-B27 positive, and sleep total score exhibited significant positive correlations (P < 0.01), as shown in **Fig 1**.

### Multivariate analysis of sleep total score in AS patients

The results in **Table 4** showed that in the multiple linear regression model with the sleep total score of AS patients as the dependent variable, a total of 12 variables entered the regression equation. The R-squared value of the model was 0.755, indicating that the age, disease duration, SF-36 scale total score, nocturnal pain VAS score, total back pain VAS score, peripheral joint pain VAS score, total fatigue score, total anxiety score, depression conversion, BASDAI score, BASFI score, and BASMI total score could explain 75.5% of the variation in the sleep total score. The model passed the F-test (F = 45.334, P ＜ 0.001). According to the overall results analysis, it was evident that the age, disease duration, nocturnal pain VAS score, total back pain VAS score, peripheral joint pain VAS score, total fatigue score, total anxiety score, and BASMI total score could have a significant positive impact on the sleep total score. However, the SF-36 scale total score, depression conversion, BASDAI score, and BASFI score did not have an impact on the sleep total score.

**Table 4:** Multivariate analysis of sleep total score in AS patients (n = 205).

	USC		SC	*t*	*p*	CD	
	*B*	SE	*Beta*		VIF	T	
Constant	‒7.107	2.368	–	‒3.001	0.003	–	–
Age	0.846	0.496	0.073	1.704	0.090	1.421	0.704
Disease duration	0.246	0.201	0.051	1.224	0.222	1.350	0.741
SF-36 total scores	‒0.007	0.011	‒0.027	‒0.596	0.552	1.641	0.610
Nocturnal pain (VAS)	0.156	0.087	0.095	1.782	0.076	2.205	0.453
Total back pain (VAS)	0.271	0.106	0.163	2.560	0.011	3.148	0.318
Peripheral joint pain (VAS)	0.366	0.100	0.208	3.670	＜0.001	2.509	0.399
Fatigue total score	0.141	0.022	0.248	6.373	＜0.001	1.182	0.846
Anxiety total score	0.044	0.019	0.135	2.326	0.021	2.630	0.380
Depression conversion	0.051	1.680	0.002	0.030	0.976	2.084	0.480
BASDAI	‒0.138	0.146	‒0.052	‒0.951	0.343	2.306	0.434
BASFI	0.110	0.137	0.044	0.805	0.422	2.339	0.428
BASMI total score	0.328	0.073	0.219	4.468	＜0.001	1.876	0.533
HLA-B27 positive	2.428	0.629	0.153	3.863	＜0.001	1.223	0.818
R^2^	0.755						
Adjust R^2^	0.739						
F	*F* (13,191) =45.334, *p* ＜ 0.001						
D-W	1.374						

*P < 0.05, **P < 0.01, USC, Unstandardized Coefficients; SC, Standardized Coefficients; CD, Collinearity Diagnosis; T, Tolerability; SE, Standard Error. Collinearity diagnostics: Variance Inflation Factor (VIF) values below 10 and Tolerability (T) values above 0.1 suggest no significant multicollinearity. Durbin-Watson statistic: D-W = 1.374 (acceptable range: 1.5–2.5), indicating no significant autocorrelation in residuals.

## Discussion

AS patients frequently experience sleep disorders, which are closely associated with disease activity, progressive deterioration of function, and fatigue [39]. This study evaluated sleep quality in AS patients using the PSQI, revealing that 66.8% of patients experienced sleep disorders, with a mean total sleep score of 8.82 ± 4.15, consistent with previous studies [40,41]. Age and disease duration were significantly associated with sleep disturbances, while gender, ethnicity, family relationships, and socioeconomic factors showed no significant correlation. Pain, fatigue, anxiety, depression, disease activity (BASDAI), functional ability (BASFI), and metrology (BASMI) were positively correlated with sleep disorders, whereas quality of life exhibited a significant negative correlation. Multivariate analysis further identified quality of life, pain, fatigue, depression, BASDAI, BASFI, BASMI, age, and disease duration as significant predictors of sleep disturbances.

With increasing age, the likelihood of experiencing sleep disorders rises, including changes in both the quality and duration of sleep [42]. According to current diagnostic guidelines, up to 20% of people meet the criteria for sleep disorders [43]. This study demonstrated that both increasing age and prolonged disease duration are independently associated with a higher prevalence of sleep disturbances in patients with AS. The positive correlation between advancing age and sleep disorders, confirmed through Pearson correlation and multiple logistic regression analyses, may be attributed to age-related declines in neural regulatory functions governing sleep-wake cycles. Similarly, longer disease duration likely exacerbates sleep disturbances through mechanisms involving chronic inflammatory pain and psychological stress. Given the frequent underassessment of sleep problems in clinical practice, these findings underscore the importance of implementing early and routine sleep evaluations, especially for older patients and those with long-standing AS, to facilitate timely interventions that may improve disease management and quality of life.

Anxiety and depression are commonly coexisting psychological disorders with physical illnesses [44], significantly impacting individuals’ behavior, emotions, and physical health [45]. Previous studies have clearly indicated that depression and anxiety are among the most common psychological disorders in AS patients [46], with a notably elevated risk of these conditions compared to the general population [47]. Clinical research has further highlighted that AS patients, particularly those with joint stiffness, often exhibit pronounced anxiety and depression symptoms [48]. Yüce et al. reported that AS patients with poor sleep quality scored significantly higher on depression and anxiety scales than those with good sleep quality [49]. Similarly, a single-center cohort study found that female AS patients with more severe sleep disturbances, as measured by the Regensburg Insomnia Scale (RIS), also displayed higher depression scores [20]. In this study, anxiety and depression were categorized into four severity levels, and revealing significant differences in sleep total scores across these groups. Multivariate logistic regression analysis identified depression as an independent risk factor associated with sleep disorders. The possible reason for this phenomenon is that psychological distress, such as depression and anxiety, may contribute to emotional instability, leading to difficulties in falling asleep, fragmented sleep, or early awakenings, which could eventually develop into chronic sleep disorder. Conversely, poor sleep quality may exacerbate psychological symptoms, creating a bidirectional relationship. Given these findings, clinicians should consider incorporating psychological assessment scales into routine evaluations to monitor patients’ mental health status. Individualized prevention and treatment plans should be formulated based on patients’ clinical characteristics, with available interventions such as pharmacological therapy [50], physical therapy [51], music therapy [52], and cognitive-behavioral therapy [53]. Additionally, family members should remain attentive to changes in the patient’s psychological state, maintain open communication with healthcare providers, and engage patients in supportive activities (e.g., conversation or recreational games) to alleviate anxiety and depression, thereby improving overall well-being and social adaptability.

Pain is the most common symptom in AS patients. A systematic review and meta-analysis encompassing 25 studies consistently demonstrated a positive correlation between pain scores and sleep disturbance scores. Notably, two of these studies specifically identified pain as a significant independent factor affecting total sleep scores [54]. In our study, we used VAS to assess the nocturnal pain, total back pain, and peripheral joint pain in AS patients. The results shown that patients with more severe sleep disorder exhibited higher total pain scores, fully demonstrating the interaction between sleep disorders and pain. Multivariate logistic regression analysis identified nocturnal pain, total back pain, and peripheral joint pain as significant risk factors for sleep disorders. This association may be mediated through elevated inflammatory markers in AS patients that exacerbate pain perception. Consequently, clinicians should carefully evaluate the pain-sleep relationship in AS patients and consider appropriate anti-inflammatory medications, analgesics, or physical therapy modalities to improve sleep outcomes.

Disease activity index is a crucial indicator for assessing the severity of AS patients. Previous studies have clarified that AS patients often exhibit high disease activity [55]. Leverment et al. conducted a systematic review comparing the prevalence and sleep disorders related factors of AS patients and non-radiographic axial spondyloarthritis. Their analysis of six included studies revealed a significant association between elevated BASDAI scores and impaired sleep quality [54]. Supporting these findings, Chen et al. reported positive correlations between sleep scores and both BASFI and BASMI, suggesting a positive association between sleep disorders in AS patients and functional ability and physical activity [18]. Our study similarly revealed positive correlation between BASDAI, BASMI, BASFI, and total sleep scores. Multivariate analysis identified BASDAI and BASMI as important risk factors for sleep disorders. These findings underscore the need for vigilant monitoring of sleep patterns in AS patients with elevated BASDAI or BASMI scores.

AS patients often experience substantial impairments in health-related quality of life (HRQoL) due to chronic pain, functional limitations, and psychological comorbidities. Yang et al.‘s systematic evaluation using the SF-36 questionnaire documented significant HRQoL reductions across all domains in AS patients [56]. Our study further revealed that AS patients with good quality of life had significantly lower total sleep scores compared to those with poor quality of life. Multivariate analysis identified HRQoL as an independent risk factor for sleep disturbances. Given the chronic, relapsing nature of AS, regular HRQoL assessments are recommended. Early interventions combining pharmacotherapy with structured physical activity programs may mitigate HRQoL deterioration, thereby reducing risks of incident psychological comorbidities and secondary sleep disorders.

Our study confirms the high prevalence of sleep disturbances in patients with AS. Multivariate analysis identified BASMI, peripheral joint pain, fatigue, anxiety, and HLA-B27 positivity as independent predictors of sleep disturbances. Notably, the predictive value of spinal mobility remained significant after adjusting for disease activity. These findings suggest that clinical evaluation of sleep problems in AS patients should particularly focus on spinal mobility, peripheral joint pain severity, and fatigue levels. Our results advance current understanding of sleep disturbance determinants in AS by demonstrating factors beyond inflammatory activity, thereby providing an evidence-based framework for identifying high-risk patients. We recommend enhanced sleep monitoring and targeted interventions for patients exhibiting these characteristics.

This study identified independent predictors of sleep disturbances in patients with AS. However, several limitations should be noted. First, the cross-sectional design precludes causal inferences regarding the observed associations, necessitating longitudinal studies to validate the temporal relationships of these predictive factors. Second, although the PSQI is a well-validated instrument, the absence of both objective sleep monitoring (e.g., polysomnography) and pulmonary function tests (PFT) may limit the accuracy of findings, particularly in characterizing sleep microstructure and its potential relationship with respiratory function. Third, the lack of systematic data on treatment regimens (e.g., biologic therapy), detailed sleep patterns (e.g., 2-week sleep diaries), and other lifestyle factors (e.g., caffeine intake, screen time) introduces potential confounding effects. Additionally, the single-center design and relatively limited sample size may constrain the generalizability of the results. To address these limitations, future research should: (1) adopt multicenter designs to enhance sample size and representativeness; (2) integrate standardized sleep diaries with objective monitoring tools (e.g., actigraphy and polysomnography) combined with PFT for comprehensive sleep assessment; (3) systematically document medication use to evaluate its modulatory effects on sleep parameters; (4) investigate the biological mechanisms underlying the association between spinal mobility (BASMI) and sleep quality; and (5) develop personalized interventions based on risk prediction models. These advancements would provide a more robust understanding of the pathophysiology of sleep disturbances in AS and inform evidence-based clinical management strategies.

## Conclusion

This study demonstrates a high prevalence of sleep disturbances in patients with AS. BASMI, peripheral joint pain, fatigue, anxiety, and HLA-B27 positivity were identified as independent predictive factors. These findings support the incorporation of spinal mobility assessment and targeted pain/fatigue management into routine sleep monitoring for AS patients. Further validation through multicenter studies incorporating objective sleep measures is warranted.

## Supporting information

S1 File Study questionnaire and scare. (XLSX)

S1 Data Original Dataset: Sleep disorders in ankylosing spondylitis patients. (DOCX)
