# Supplementary material for: Analysis of sleep disorders and their influencing factors in patients with ankylosing spondylitis
Source: PLoS One. 2025 May 13;20(5):e0323324. doi: 10.1371/journal.pone.0323324 (PMC12074546; doi:10.1371/journal.pone.0323324)
Supplement: S1 Data — (DOCX) [file pone.0323324.s002.docx]

**S Table1: Basic Information Survey for Patients with Ankylosing Spondylitis**

Hello!

To better understand the current status of sleep disorders in patients with ankylosing spondylitis and identify the related influencing factors, we are conducting this questionnaire survey. Please answer the questions based on your current situation by marking the corresponding options or filling in the blanks. Kindly complete all the questions without omissions. The information you provide will only be used for research purposes and will not be disclosed to any third parties. Thank you for your cooperation!

Name:

1. Gender: Male □ Female □
2. Date of Birth:
3. Age:
4. Ethnicity: Han □ Other □
5. Are you an only child? Yes □ No □
6. Occupation: Worker □ Farmer □ Employee in a public institution or enterprise □ Self-employed or freelancer □ Student □ Unemployed □
7. Family structure: Nuclear family □ Extended family □ Reorganized/single-parent family □
8. Education level: Junior high school or below □ High school/vocational school □ College/associate degree □ Graduate degree or above □
9. Disease duration (in years):

10. Is HLA-B27 positive? Yes □ No □

**S Table2 Short Form (36) Health Survey**

| **Item Reponse option (score)** | |
| --- | --- |
| (1) In general, would you say your health is:  (2) Compared to one year ago, how would you rate your health in general now? | Excellent (4) Very good (3) Good (2)  Fair (1) Poor (0)  Much better now than one year ago (*)  Somewhat better now than one year ago (*) About the same (*)  Somewhat worse now than one year ago (*) Much worse now than one year ago (*) |
| *The following items are about activities you might do during a typical day. Does your health now limit you in these activities? If so, how much?*  (3) Vigorous activities, such as running, lifting Yes, limited a lot (0) heavy objects, participating in strenuous sports Yes, limited a little (1)  (4) Moderate activities, such as moving No, not limited at all (2)  a table, pushing a vacuum cleaner, bowling, or playing golf  (5) Lifting or carrying groceries  (6) Climbing several ﬂights of stairs  (7) Climbing one ﬂight of stairs  (8) Bending, kneeling, or stooping  (9) Walking more than a mile  (10) Walking several blocks  (11) Walking one block  (12) Bathing or dressing yourself  *During the past 4 weeks, have you had any of the following problems with your work or other regular daily activities as a result of your physical health?* | |
| (13) Cut down the amount of time you spent on work or other activities  (14) Accomplished less than you would like  (15) Were limited in the kind of work or other activi- ties  (16) Had difﬁculty performing the work or other ac- tivities (for example, it took extra effort) | Yes (0) No (1) |

*Continued on next page*

| **Item Reponse option (score)** | | | |
| --- | --- | --- | --- |
| *During the past 4 weeks, have you had any of the following problems with your work or other regular daily activities as a result of any emotional problems (such as feeling depressed or anxious)?* | | | |
| (17) Cut down the amount of time you spent on work or other activities  (18) Accomplished less than you would like  (19) Didn’t do work or other activities as carefully as usual  (20) During the past 4 weeks, to what extent has your physical health or emotional problems  interfered with your normal social activities with  family, friends, neighbors, or groups?  (21) How much bodily pain have you had during the past 4 weeks?  (22) During the past 4 weeks, how much did pain interfere with your normal work (including both  work outside the home and housework)? | | Yes (0) No (1)  Not at all (4)  Slightly (3)  Moderately (2) Quite a bit (1) Extremely (0)  None (5)  Very mild (4) Mild (3)  Moderate (2)  Severe (1)  Very severe (0)  Not at all (4)  A little bit (3)  Moderately (2) Quite a bit (1) Extremely (0) | |
| *These questions are about how you feel and how things have been with you during the past 4 weeks. For each question, please give the one answer that comes closest to the way you have been feeling. How much of the time during the past 4 weeks...* | | | |
| (23) Did you feel full of pep?  (24) Have you been a very nervous person?  (25) Have you felt so down in the dumps that nothing could cheer you up?  (26) Have you felt calm and peaceful?  (27) Did you have a lot of energy?  (28) Have you felt downhearted and blue?  (29) Did you feel worn out?  (30) Have you been a happy person?  (31) Did you feel tired?  (32) During the past 4 weeks, how much of the time has your physical health or emotional problems interfered with your social activities (like visiting with friends, relatives, etc.)? | All of the time (0)  Most of the time (1)  A good bit of the time (2) Some of the time (3)  A little of the time (4) None of the time (5)  All of the time (0)  Most of the time (1) Some of the time (2) A little of the time (3) None of the time (4) | | |
| *How TRUE or FALSE is each of the following statements for you.*  (33) I seem to get sick a little easier than other peo- ple  (34) I am as healthy as anybody I know  (35) I expect my health to get worse  (36) My health is excellent | | | Deﬁnitely true (0)  Mostly true (1)  Don’t know (2)  Mostly false (3)  Deﬁnitely false (4) |

**S Table3 Pittsburgh Sleep Quality Index (PSQI)**


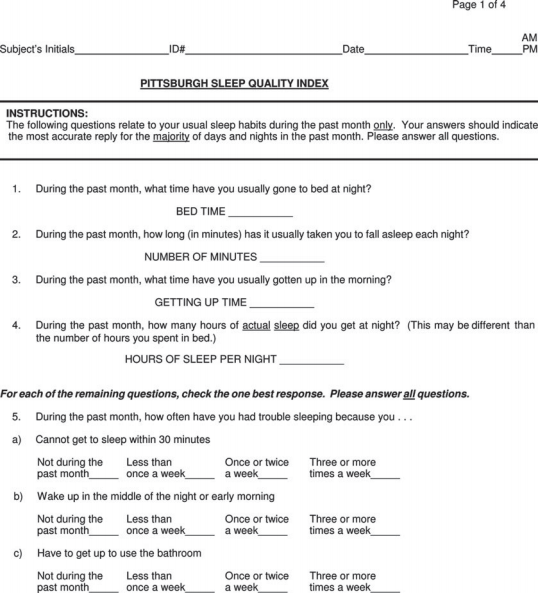


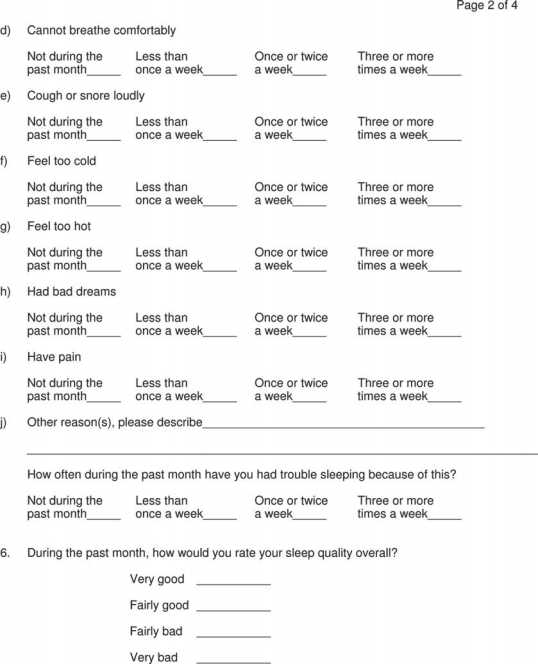


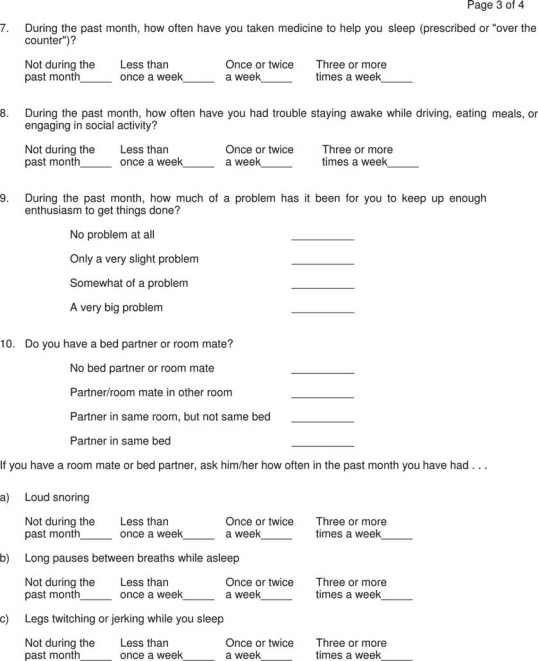


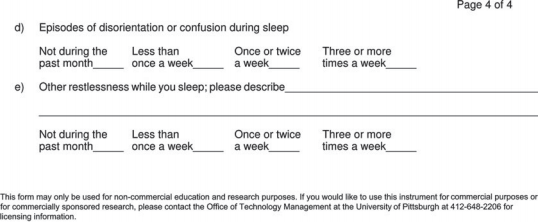


**S Table4 Visual Analogue Scale (VAS)**

**
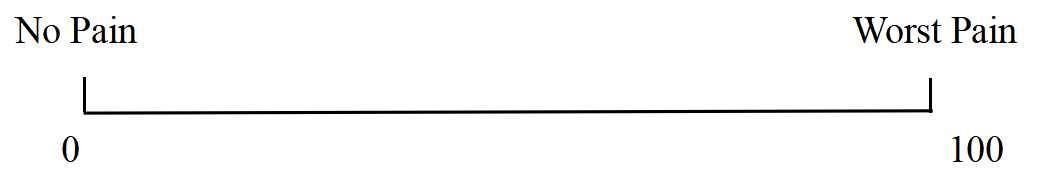
**

The left end represents "No Pain," the middle represents varying levels of pain, and the right end represents "Worst Pain" (Pain intensity increases gradually from left to right).

Please mark a vertical line "│" on the horizontal line to indicate the intensity of your current pain based on night pain, overall back pain, and peripheral joint pain in the past week.

1. **Night Pain VAS**

**
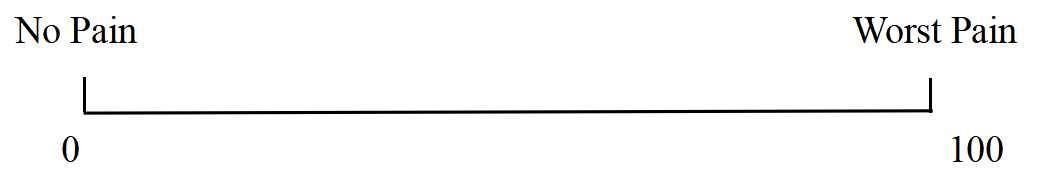
**

1. **Overall Back Pain VAS**


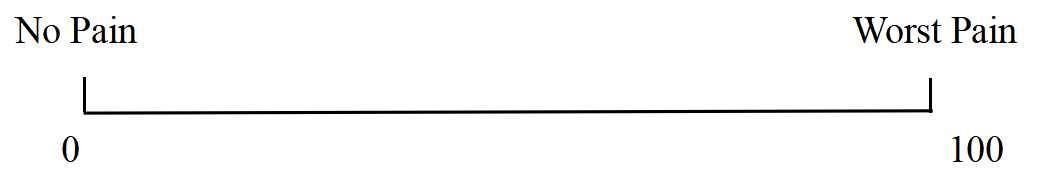


1. **Peripheral Joint Pain VAS**


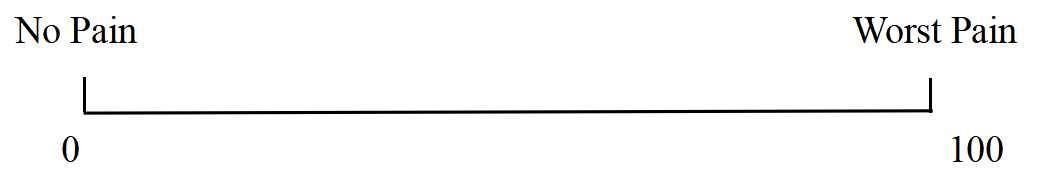


**S Table5 Multidimensional Fatigue Inventory (MFI)**


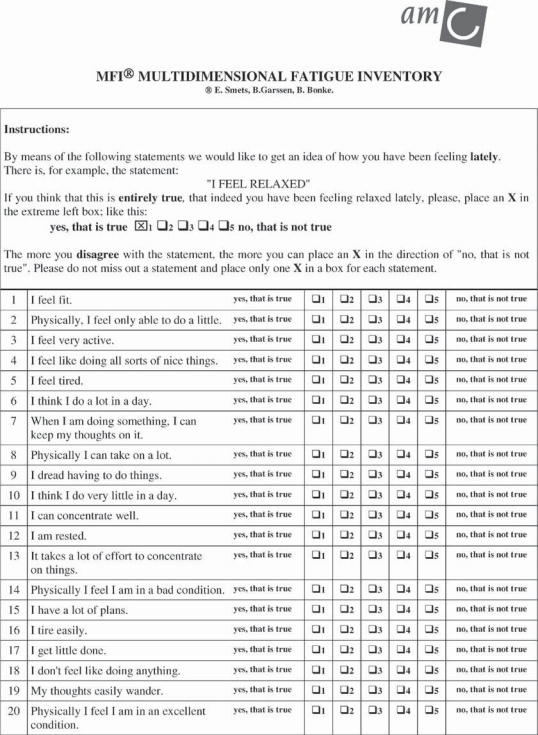


**S Table6 Zung Self-Rating Anxiety Scale**

| how you have been feeling **during the past week**. Circle the appropriate number for each statement. | **None or a little of the time** | **Some of the time** | **Good**  **part of the time** | **Most or**  **all of the**  **time** |
| --- | --- | --- | --- | --- |
| 1. I feel more nervous and anxious than usual. | 1 | 2 | 3 | 4 |
| 2. I feel afraid for no reason at all. | 1 | 2 | 3 | 4 |
| 3. I get upset easily or feel panicky. | 1 | 2 | 3 | 4 |
| 4. I feel like I'm falling apart and going to pieces. | 1 | 2 | 3 | 4 |
| 5. I feel that everything is all right and nothing bad will happen. | 4 | 3 | 2 | 1 |
| 6. My arms and legs shake and tremble. | 1 | 2 | 3 | 4 |
| 7. I am bothered by headaches, neck and back pains. | 1 | 2 | 3 | 4 |
| 8. I feel weak and get tired easily. | 1 | 2 | 3 | 4 |
| 9. I feel calm and can sit still easily. | 4 | 3 | 2 | 1 |
| 10. I can feel my heart beating fast. | 1 | 2 | 3 | 4 |
| 11. I am bothered by dizzy spells. | 1 | 2 | 3 | 4 |
| 12. I have fainting spells or feel faint. | 1 | 2 | 3 | 4 |
| 13. I can breathe in and out easily. | 4 | 3 | 2 | 1 |
| 14. I get feelings of numbness and tingling in my fingers and toes. | 1 | 2 | 3 | 4 |
| 15. I am bothered by stomachaches or indigestion. | 1 | 2 | 3 | 4 |
| 16. I have to empty my bladder often. | 1 | 2 | 3 | 4 |
| 17. My hands are usually dry and warm. | 4 | 3 | 2 | 1 |
| 18. My face gets hot and blushes. | 1 | 2 | 3 | 4 |
| 19. Ifall asleep easily and get a good night's rest. | 4 | 3 | 2 | 1 |
| 20. I have nightmares. | 1 | 2 | 3 | 4 |

**S Table7 Zung Self-Rating Depression Scale (SDS)**

For each item below, please place a check mark (
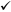
) in the column which best describes how often you felt or behaved this way during the past several days

| **Place check mark (**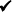 **) in correct column.** | **A little of the time** | **Some of the time** | **Good part of the time** | **Most of the time** |
| --- | --- | --- | --- | --- |
| 1. I feel down-hearted and blue. |  |  |  |  |
| 2. Morning is when I feel the best. |  |  |  |  |
| 3. I have crying spells or feel like it. |  |  |  |  |
| 4. I have trouble sleeping at night. |  |  |  |  |
| 5. I eat as much as I used to. |  |  |  |  |
| 6. I still enjoy sex. |  |  |  |  |
| 7. I notice that I am losing weight. |  |  |  |  |
| 8. I have trouble with constipation. |  |  |  |  |
| 9. My heart beats faster than usual. |  |  |  |  |
| 10. I get tired for no reason. |  |  |  |  |
| 11. My mind is as clear as it used to be. |  |  |  |  |
| 12. I find it easy to do the things I used to. |  |  |  |  |
| 13. I am restless and can’t keep still. |  |  |  |  |
| 14. I feel hopeful about the future. |  |  |  |  |
| 15. I am more irritable than usual. |  |  |  |  |
| 16. I find it easy to make decisions. |  |  |  |  |
| 17. I feel that I am useful and needed. |  |  |  |  |
| 18. My life is pretty full. |  |  |  |  |
| 19. I feel that others would be better off if I were dead. |  |  |  |  |
| 20. I still enjoy the things I used to do. |  |  |  |  |

**S Table8 Bath Ankylosing Spondylitis Disease Activity Index (BASDAI)**

**S Table9 Bath Ankylosing Spondylitis Functional Index (BASFI)**

Marquerd’un trait laréponse à chacune des questions en vous référant aux dernières 48 heures.

1. Pouvez-vous mettre vos chaussettes ou collants sans l’aidedequelqu’un ou de tout autre moyen extérieur (ex. : petit appareil vous aidant à mettre leschaussettes) ?

*Sans aucune difficulté*
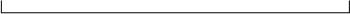
 *Impossible*

2. Pouvez-vous vous pencher en avant pour ramasser un stylo posé sur le sol sans l’aided’un moyen extérieur ?

*Sans aucune difficulté*
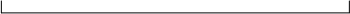
 *Impossible*

3. Pouvez-vous atteindre une étagère élevée sans l’aidedequelqu’un ou d’un moyen extérieur ?

*Sans aucune difficulté*
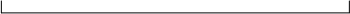
 *Impossible*

4. Pouvez-vous vous lever d’une chaise sans accoudoir sans utiliser vos mains ou toute autre aide ?

*Sans aucune difficulté*
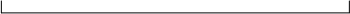
 *Impossible*

5. Pouvez-vous vous relever dela position « couché sur ledos », sans aide ?

*Sans aucune difficulté*
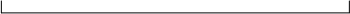
 *Impossible*

6. Pouvez-vous rester debout sans soutien pendant 10 minutes sans ressentir de gêne ?

*Sans aucune difficulté*
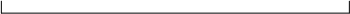
 *Impossible*

7. Pouvez-vous monter 12 à 15 marches, en ne posant qu’un pied sur chaque marche, sans vous tenir à la rampe ou utiliser tout autre soutien ?

*Sans aucune difficulté*
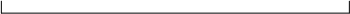
 *Impossible*

8. Pouvez-vous regarder par-dessus votre épaule sans vous retourner ?

*Sans aucune difficulté*
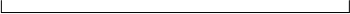
 *Impossible*

9. Pouvez-vous effectuer des activités nécessitant un effort physique (ex : mouvements dekinésithérapie, jardinage ou sports) ?

*Sans aucune difficulté*
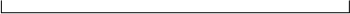
 *Impossible*

10. pouvez-vous avoir des activités toute la journée, que ce soit au domicile ou au travail ?

*Sans aucune difficulté*
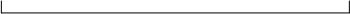
 *Impossible*

**S Table10 Bath Ankylosing Spondylitis Metrology Index (BASMI)**

| **Lateral lumbar flexion**: Patient stands with heels and buttocks touching the wall, knees straight, shoulders  Assessment: Score:  back, hands by the side. The patient is then asked to bend to the right side as far as possible without lifting the left foot/heel or flexing the right knee, and maintaining a straight posture with heels, buttocks, and shoulders against the wall. The distance from the third fingertip to the floor when patient bends to the side, is subtracted from the distance when patient stands upright. The manoeuvre is repeated on the left side.   \| > 20 0 \| 18–20 1 \| 15,9–18,9 2 \| 13,8–15,8 3 \| 11,7–13,79 4 \| 9,6–11,6 5 \| 7,5–9,5 6 \| 5,4–7,4 7 \| 3,3–5,3 8 \| 1,2–3,2 9 \| < 1,2 10 \| \| --- \| --- \| --- \| --- \| --- \| --- \| --- \| --- \| --- \| --- \| --- \| |
| --- | --- | --- | --- | --- | --- | --- | --- | --- | --- | --- | --- |

| **Tragus-to-wall distance**: Maintain same starting position as above. Ensure head in as neutral position (ana-  tomical alignment) as possible, chin drawn in as far as possible. Measure distance between tragus of the ear  and wall on both sides, using a rigid ruler. Ensure no cervical extension, rotation, flexion or side flexion occurs. | |
| --- | --- |
| Assessment: Score: | < 10 10–12 13–15 16–18 19–21 22–24 25–27 28–30 31–33 34–36 > 36  0 1 2 3 4 5 6 7 8 9 10 |

| **Lumbar flexion (modified Schober)**: With the patient standing upright, place a mark at the lumbosacral  Assessment: Score:  junction (at the level of the dimples of Venus on both sides). Further marks areplaced 5 cm below and 10 cm above 1. Measure the distraction of these two marks when the patient bends forward as far as possible, keeping the kneestraight.  1) Among the "modified Schober"s published in the literature, the modification recommended by Macrae and Wright is used.   \| > 7,0 0 \| 6,4–7,0 1 \| 5,7–6,3 2 \| 5,0–5,6 3 \| 4,3–4,9 4 \| 3,6–4,2 5 \| 2,9–3,5 6 \| 2,2–2,8 7 \| 1,5–2,1 8 \| 0,8–1,4 9 \| < 0,8 10 \| \| --- \| --- \| --- \| --- \| --- \| --- \| --- \| --- \| --- \| --- \| --- \| |
| --- | --- | --- | --- | --- | --- | --- | --- | --- | --- | --- | --- |

| **Maximal intermalleolar distance:** Patient supine on the floor or a wide plinth, with the knees straight and the feet pointing straight up. Patient is asked to separate legs along the resting surface as far as possible. Distance between medial malleoli is measured.  Assessment: Score:   \| > 119 0 \| 110–119 1 \| 100–109 2 \| 90–99 3 \| 80–89 4 \| 70–79 5 \| 60–69 6 \| 50–59 7 \| 40–49 8 \| 30–39 9 \| < 30 10 \| \| --- \| --- \| --- \| --- \| --- \| --- \| --- \| --- \| --- \| --- \| --- \| |
| --- | --- | --- | --- | --- | --- | --- | --- | --- | --- | --- | --- |

| **Cervical rotation:** Patient supine on plinth, head in neutral position, forehead horizontal (if necessary head on pillow or foam block to allow this, must be documented for future reassessments). Gravity goniometer placed centrally on the forehead. Patient rotates head as far as possible, keeping shoulders still, ensure no neck flexion or side flexion occurs.   \| > 85,0 0 \| 76,6–85 1 \| 68,1–76,5 2 \| 59,6–68,0 3 \| 51,1–59,5 4 \| 42,6–51,0 5 \| 34,1–42,5 6 \| 25,6–34,0 7 \| 17,1–25,5 8 \| 8,6–17,0 9 \| < 8,6 10 \| \| --- \| --- \| --- \| --- \| --- \| --- \| --- \| --- \| --- \| --- \| --- \| |
| --- | --- | --- | --- | --- | --- | --- | --- | --- | --- | --- | --- |
